# Fig Tree-Induced Phytophotodermatitis: A Case Report on the Perils of a Hobby

**DOI:** 10.7759/cureus.41888

**Published:** 2023-07-14

**Authors:** Ana Raquel Pinto, Inês Machado Cunha, Eva Rebelo Gomes

**Affiliations:** 1 Allergy and Immunology Department, Centro Hospitalar Universitário de Santo António, Porto, PRT

**Keywords:** phytochemicals, photosensitivity disorders, phytophotodermatitis, furocoumarins, fig tree

## Abstract

Phytophotodermatitis, a condition that results from sequential skin exposure to phototoxic chemicals contained within plants, followed by exposure to solar ultraviolet radiation, has been described with several plants and plant-based foods, namely members of the *Moraceae*family, which include *Ficus carica *L. This tree’s branches, leaves, and fruit skin exude a milky sap or latex containing proteolytic enzymes and furocoumarins known to be photoirritants, easily absorbed upon skin contact. Oxygen-dependent and independent toxic reactions subsequent to sun exposure promote cell membrane damage and oedema, consequently leading to cell death. The diagnosis is confirmed with a detailed anamnesis, and photopatch testing is often useful to rule out a differential diagnosis. It is typically a self-limited condition, with few cases requiring treatment with topical or systemic corticosteroids. We report on a 55-year-old male patient who, following picking figs and pruning a fig tree while exposed to sunlight, developed erythematous and pruritic maculopapular lesions that progressed to blisters with residual hyperpigmentation. The diagnosis was further corroborated through photopatch testing, and the patient was recommended to avoid this recreational activity without symptoms’ relapse. This case highlights the importance of considering phytophotodermatitis as a differential diagnosis when evaluating cases of dermatitis on exposed body surfaces and the importance of an exhaustive anamnesis. Identification of specific plant triggers and the performance of photopatch tests are essential to help confirm the diagnosis and guide avoidance recommendations.

## Introduction

Phytophotodermatitis is a cutaneous reaction that results from sequential skin exposure to phototoxic chemicals contained within plants, such as furocoumarins, followed by exposure to solar ultraviolet (UV) radiation. Several plants and plant-based foods have already been recognized as implicated, namely members of the *Moraceae* family, which include *Ficus carica* L. (fig tree) [1].

The classic patient presents in the summer or following a vacation period when there is a higher furocoumarins’ concentration, as well as other factors that enhance photoreactivity, such as greater sunlight, heat, and environmental humidity [2]. Typical manifestations include burning erythema and edema with a linear distribution that may subsequently blister, and post-inflammatory hyperpigmentation lesions lasting months to years [1]. This diagnosis may follow recreational or occupational exposure to plants or plant-based food, namely by gardeners, landscapers, and farmers.

Phytophotodermatitis is frequently confused with a broad spectrum of dermatological conditions, namely sun or chemical burns, drug hypersensitivity, and atopic or contact dermatitis. An important differential diagnosis is photoallergic dermatitis, and differentiation between these two entities is not always easy as clinical presentations may overlap. Photoallergic dermatitis is a delayed-type hypersensitivity reaction to an allergen whose antigenicity increases following solar UV exposure. This disorder, which requires previous sensitization to the suspected allergen, results in pruritic and eczematous acute, subacute, or chronic lesions in exposed and sometimes unexposed skin, peaking at 24 hours following sun exposure. Usually, there are no associated residual lesions or pigmentary changes. Photopatch testing with the suspected substance is often useful during the investigation, confirming UV radiation's contribution to the pathophysiology of the skin lesions as well as the suspected plant's or plant-derived food’s involvement [3-5].

We present a case report of a male patient who was diagnosed with fig-tree-induced phytophotodermatitis following recreational exposure to this tree and its fruits and the investigation that led to the diagnosis.

## Case presentation

**Figure 1:**
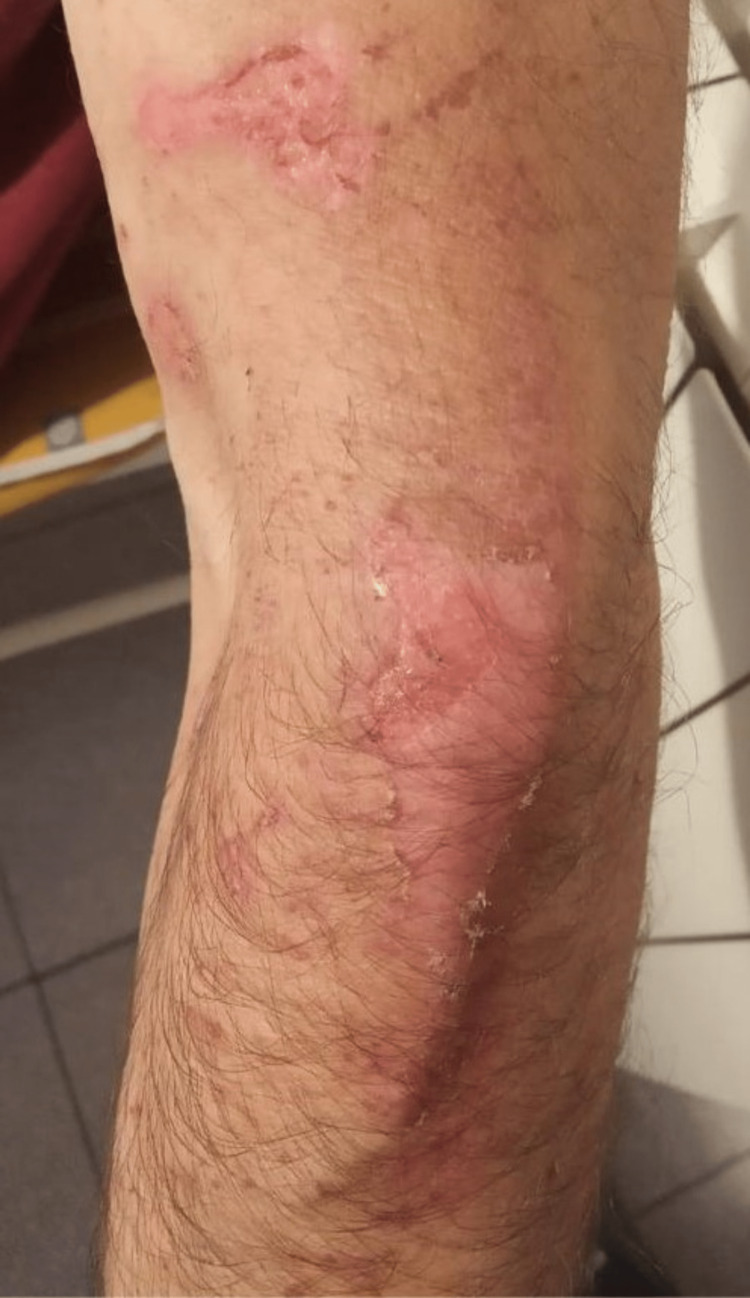
Skin lesions Erythematous and desquamative lesions, in conjunction with scratching abrasions, are visible on the posterior aspect of the patient's upper arm and forearm.

**Figure 2:**
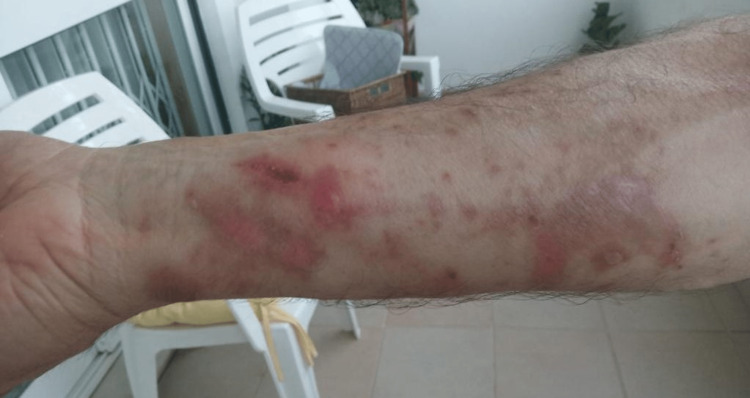
Skin lesions The affected area exhibits a xerotic appearance, along with erythematous lesions and crusting lesions, which subsequently result in residual hyperpigmentation.

A 55-year-old male patient, with no relevant personal history or regular medication, was evaluated following two distinct but similar episodes with the development of erythematous, mildly painful, and pruritic maculopapular lesions on both upper arms and forearms. These lesions progressed to blisters in the course of 12 hours and resolved with residual hyperpigmentation one week after onset (Figure 1 and Figure 2). There were no other associated signs or symptoms. Contact with drugs, food, or different hygiene or cosmetic products was excluded. The patient mentioned that episodes coincided with sunny days when he was picking figs and pruning a fig tree, recognizing that lesions only developed on exposed body areas.

Following referral to the allergy clinic, photopatch tests were performed with a locally made preparation created from the maceration of fig tree leaves and stems. The preparation was placed on both forearms, and contact was maintained for one hour. The patient was then asked to keep the area in contact with the preparation uncovered during the day, only on one of the forearms, allowing sun exposure. About eight hours later, he reported discomfort and erythema at the site of contact with the preparation, only on the forearm exposed to solar radiation, with no complaints in the unexposed arm. The reaction’s reproducibility only on the forearm exposed to solar radiation, along with a suggestive anamnesis, confirmed the diagnosis of phytophotodermatitis triggered by the fig tree. The patient received guidance to prevent future direct contact with fig trees and their fruits, particularly during periods of heightened sun exposure, and to wear protective clothing, such as long-sleeved shirts, long pants, and gloves, when necessary. Additionally, emergency topical medication was provided to the patient in the event of accidental reexposure or recurrence of lesions.

## Discussion

*Ficus carica* L.’s branches, leaves, and fruit skin, when cut, exude a milky sap or latex containing proteolytic enzymes and linear furocoumarins (psoralen and bergapten) that are known to be photoirritants and also potential photosensitizers [3]. Their photochemical excitation is induced by UV radiation, typically within the UVA wavelength range of 320-400 nm. Two types of toxic reactions occur: an oxygen-independent reaction where the UV-activated furocoumarins bind to RNA and nuclear DNA, and an oxygen-dependent reaction where the induced compounds cause cell membrane damage and oedema, consequently leading to cell death (sunburnt cells and apoptotic keratinocytes) [6].

Phytophotodermatitis, typically a self-limited condition without long-term sequelae, does not currently have established treatment guidelines. The traditional approach focuses on symptomatic treatment and future avoidance of the suspected trigger, namely through protective clothing. However, moderately severe lesions might require treatment with a topical corticosteroid, and in severe, widespread lesions, oral corticosteroids can be used. Antibiotics are reserved for bacterial superinfection, one of the possible complications. To ascertain the efficacy and safety of these management approaches, further research is needed [4,7].

## Conclusions

Although fig tree-induced phytophotodermatitis has been previously reported in the literature, it is often underdiagnosed. This may be due to its similarity in appearance to other dermatological conditions as well as a lack of awareness and recognition among healthcare professionals.

The reported case highlights the importance of a detailed anamnesis during the evaluation of cutaneous manifestations. When phytophotodermatitis is suspected, the identification of specific plant triggers and the performance of photopatch tests are essential to help confirm the diagnosis and guide avoidance recommendations.
